# Deciphering Site-Specific Regulatory Networks of the Kinesin Protein KIF21A Through Integrative Phosphoproteomic Analysis

**DOI:** 10.3390/ijms27146387

**Published:** 2026-07-18

**Authors:** Shanmitha B. Rai, Ayadathil Sujina, Mukhtar Ahmed, Sreeshma Ravindran Kammarambath, Suhail Subair, Athira Perunelly Gopalakrishnan, Apoorva Pai Kalasa Anil Kumar, Levin John, Rajesh Raju, Akhina Palollathil

**Affiliations:** 1 Centre for Integrative Omics Data Science (CIODS), Yenepoya (Deemed to be University), Mangalore 575018, India; shanmithabrai.ciods@yenepoya.edu.in (S.B.R.); sujinaa.ciods@yenepoya.edu.in (A.S.);; 2 Department of Zoology, College of Science, King Saud University, P.O. Box 2455, Riyadh 11451, Saudi Arabia; mahmed1@ksu.edu.sa; 3 Institute for Regeneration and Repair, University of Edinburgh, Edinburgh EH16 4UU, UK; vblevinjohn@gmail.com

**Keywords:** KANK, CMSC, microtubule stabilization, WD-40 repeats, KIF21A, co-regulation

## Abstract

KIF21A, a member of the Kinesin-4 family of motor proteins, is involved in the regulation of microtubule dynamics and intracellular transport, with emerging evidence suggesting its potential role in cancer progression. In this study, we performed an integrative analysis of over 3825 human phosphoproteomics studies to characterize site-specific phosphorylation of KIF21A. Three predominant phosphosites were identified in KIF21A (S853, S1212, and S1239) with the highest detection frequency across phosphoproteomics studies, and were analyzed for co-regulation patterns to identify potential kinase associations and functional networks. Phosphosite S853 showed a strong association with cytoskeletal organization and cortical microtubule stabilization complexes (CMSCs) components, including KANK1 (S186), PHLDB2 (S513, S42) and CLASP1 (S600, S572, S646), indicating its role in cytoskeletal organization. Upstream kinase analysis identified potential regulators, such as PAK2, RPS6KA1/A3, RPS6KB1, CHEK1/2 and CDK18/16 with site-specific variability in their associations with KIF21A predominant sites. Interestingly, phosphosite-specific correlation analysis between KIF21A and candidate kinases revealed that the KIF21A S1239 phosphosite exhibited tumor-specific correlations with CDK18 across multiple cancer types. Functional enrichment revealed that co-regulated phosphoproteins were involved in cytoskeleton regulation, cell cycle regulation, and carcinogenesis. Pan-cancer analysis demonstrated dysregulated expression of KIF21A in multiple tumor types, with stage-associated upregulation in selected cancers. Gene-level validation further supported these findings, showing consistent positive correlations between KIF21A and key regulators such as CTNND1 and PTK2, as well as other cytoskeleton and cancer-associated genes. Overall, this study highlights site-specific phosphorylation as a key regulatory mechanism of KIF21A and suggests its involvement in cytoskeleton-associated signaling networks in cancer.

## 1. Introduction

Kinesin superfamily proteins (KIFs) comprise a wide range of motor proteins that depend on microtubules and use ATP hydrolysis to move organelles, protein complexes, and mRNA around cells [1,2]. To date, more than 45 members in KIFs have been identified and are collectively classified into 14 distinct classes [3]. These include C-terminal kinesins, C-1 kinesin, and C-2 kinesin, a single class of M-kinesins with centrally located motor domains and 11 classes of N-terminal kinesins, which together comprise 16 distinct Kinesin families [3]. Members of the Kinesin-4 family play critical roles in cell division, microtubule organization, and signaling [4]. The Kinesin-4 family is characterized by its ability to modulate and often inhibit microtubule dynamics and is subdivided into subfamilies, such as KIF4, KIF7, KIF27, and KIF21 [1,5].

Kinesin family member 21A (KIF21A), a member of the Kinesin-4 subfamily, is characterized by an N-terminal motor domain, a coiled-coil stalk region, and C-terminal WD40 repeat domains that are involved in cargo recognition and autoregulatory interaction [1]. WD-40 repeats are known to facilitate protein–protein interactions [6]. In humans, the KIF21A gene is located on chromosome 12q12 and encodes a protein with a molecular mass of ≈187 kDa [7]. KIF21A is ubiquitously expressed in human tissues, particularly in neurons in the central and peripheral nervous systems [8]. KIF21A is primarily studied in neurons and is known to be essential for the anterograde intracellular transport in neurons [1,9]. Additionally, KIF21A is an N-Kinesin that acts as a plus-end-directed motor to move cargo away from the microtubule-organizing center [10]. A defining regulatory feature of KIF21A is its autoinhibitory regulation, through intramolecular interactions between the motor domain and specific coiled-coil regions to suppress motor activity in the absence of activation signals. Genetic and structural studies indicate that this autoinhibition is critical for normal function, and disruption of the autoinhibitory interface leads to aberrant motor activation and dysregulated microtubule dynamics [11,12].

Previous studies have discovered that mutations in KIF21A lead to a neurological disorder known as congenital fibrosis of the extraocular muscles type 1 (CFEOM1) [12]. The KIF21A mutations identified in CFEOM1 predominantly modify various conserved amino acid residues within the KIF21A third coiled-coil stalk domain [13]. The second coiled-coil domain also has a role in functional control [14]. Recently, peripheral neuropathy has been linked to p.Leu664Pro, a novel de novo missense mutation in this domain, indicating wider neurological consequences beyond CFEOM1 [14]. Although KIF21A’s structural regulation, interaction partners, and disease-associated mutations have been clarified by earlier research [11,12,13], its post-translational regulation and phosphorylation-mediated control are still unknown. Phosphorylation can influence protein function, interactions, and localization, but its site-specific regulation in KIF21A is poorly understood.

To investigate the possible phosphoregulatory network of KIF21A, we conducted an integrative analysis of publicly available phosphoproteomics datasets. By evaluating the expression pattern of KIF21A phosphosite in phosphoproteomics datasets, we identified the most common phosphorylation sites in KIF21A and examined their co-regulation patterns. Several co-regulated phosphoproteins were identified, many of which represented interacting partners with the potential to be the candidate upstream kinases and downstream substrates. Further, we also analyzed the phosphosite-specific correlations of KIF21A with upstream kinases and co-regulated proteins involved in carcinogenesis, leading to the identification of potential functional associations. These data-driven inferences may provide initial insights into site-specific regulatory mechanisms of KIF21A and could serve as a foundation for future functional studies.

## 2. Results

### 2.1. Compilation of Phosphoproteomics Datasets and Identification of Major Phosphosites of KIF21A

We examined 3825 publicly accessible human global cellular phosphoproteomics studies to identify the functionally relevant phospho-signaling patterns associated with KIF21A. The collected studies were categorized into two dataset types: qualitative profiling datasets, in which test conditions and controls were treated as independent phosphosite profiling datasets, and quantitative differential datasets, in which biological or experimental test conditions were directly compared with their corresponding controls. Using this approach, we identified 216 differential datasets and 677 profiling datasets containing information on KIF21A phosphorylation (Supplementary Tables S1 and S2). Mapping of phosphosites within KIF21A identified 51 phosphosites from profiling datasets and 26 phosphosites from differential expression datasets. Ten phosphosites (S224, T931, S1263, S1269, S1231, T1297, S530, S529, T522, and S496) out of the 51 phosphosites from profiling datasets were not yet annotated in the PhosphoSitePlus database [7].

**Figure 1:**
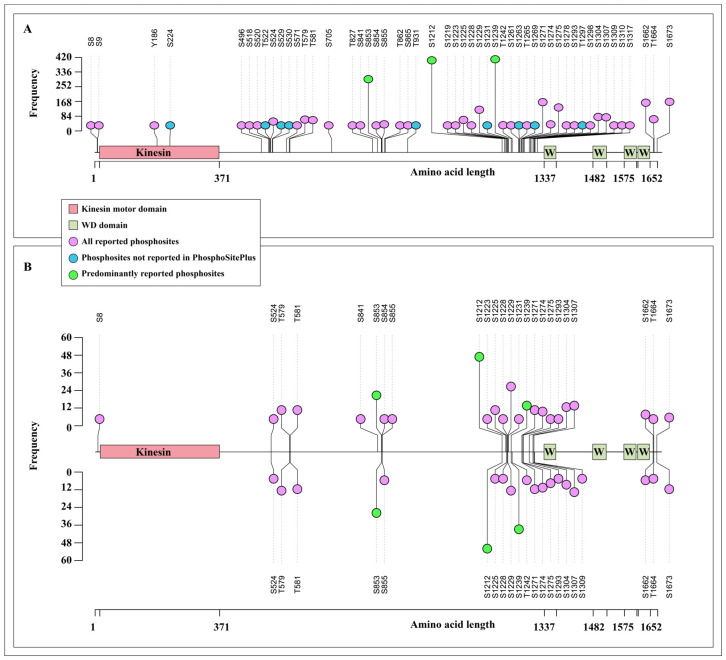
Lollipop plot illustrating the frequency of detection of phosphosites in KIF21A, with each node representing a specific phosphosite. (**A**) Class-1 phosphosites identified in KIF21A across qualitative profiling datasets. Schematic representation of KIF21A phosphosites mapped along the amino acid sequence, indicating the Kinesin motor and WD domains. The y-axis represents phosphosite frequency across analyzed datasets. Predominant phosphosites and phosphosites not previously reported in PhosphoSitePlus are highlighted. (**B**) Class-1 phosphosites identified in KIF21A across quantitative differential datasets. Phosphosites plotted above the x-axis represent positively co-regulated phosphosites, whereas those below the x-axis represent negatively co-regulated phosphosites. The y-axis indicates the frequency of phosphosite co-regulation across datasets.

Subsequently, phosphosites were prioritized based on their frequency of detection across assembled datasets. Phosphosites with the highest frequency from the profiling datasets were S1239 (frequency = 502), S1212 (frequency = 467), and S853 (frequency = 339). Similarly, in the differential datasets, S1212 (frequency = 101), S1239 (frequency = 55), and S853 (frequency = 51) showed the highest occurrence. Based on their consistent detection across datasets, these three sites were considered as the predominant phosphosites of KIF21A. Interestingly, all three predominant phosphosites are located within the central region of the protein, between the N-terminal motor domain and the C-terminal WD40 repeat domains (Figure 1).

### 2.2. Evolutionary Conservation of Predominant KIF21A Phosphosites Within the Kinesin-4 Family

In mammals, the Kinesin-4 family includes KIF7, KIF27, KIF4A, KIF4B, KIF21A, and KIF21B. Although these proteins share a high degree of sequence similarity, they perform distinct cellular functions. Members of this family have been reported to regulate important biological processes, such as cell proliferation and differentiation [15], chromosome condensation and segregation during mitosis [16], and the control of microtubule growth and pause, which is essential for proper neuronal morphology [17].

**Figure 2:**
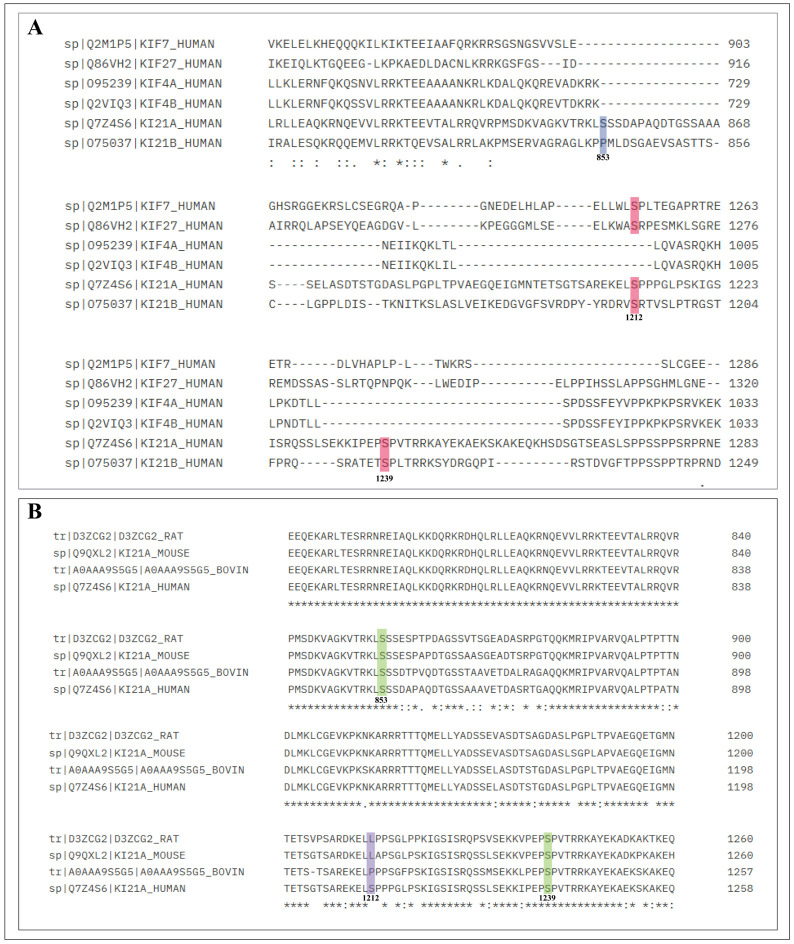
(**A**). Multiple sequence alignment of the KIF4A family, highlighting the conservation status of identified predominant phosphosites. Residues highlighted in blue indicate predominant phosphosites that are not conserved between the two proteins, whereas residues marked in red boxes represent conserved predominant phosphosites. (**B**). Ortholog analysis of KIF21A across different species. Residues highlighted in green and purple indicate conserved and non-conserved predominant phosphosite. “*” indicates conserved sequence (identical), “:” indicates conservative mutation, “.” indicates semi-consevative mutation, “-” indicates gap.

To further understand the potential functional importance of the KIF21A predominant phosphosites identified in this study, we examined their evolutionary conservation across kinesin-4 family members. Interestingly, the predominant KIF21A phosphorylation site S1239 was found to be conserved in KIF21B at S1215, and KIF21A phosphorylation site S1212 was found to be conserved in KIF21B at S1193 and was also found to be conserved in KIF7 at S1252 and KIF27 at S1265, whereas conservation was not observed in KIF4A and KIF4B indicating differential evolutionary retention of this phosphoregulatory residue among Kinesin family proteins (Figure 2A). Furthermore, orthologous conservation analysis across species revealed that the KIF21A phosphosites S853 and S1239 were conserved among the species, supporting their potential functional and evolutionary importance (Figure 2B).

### 2.3. Co-Regulation Analysis of Predominant KIF21A Phosphosites

**Figure 3:**
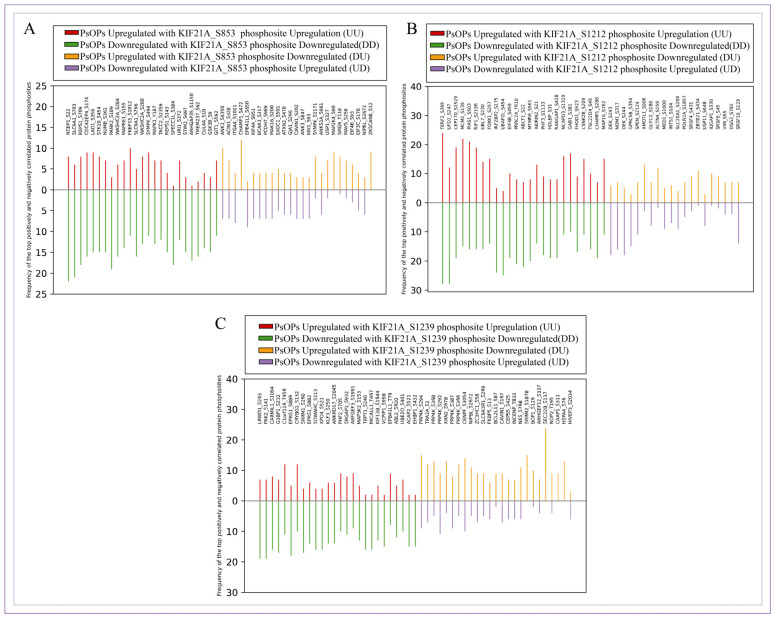
Bar plots showing the top 25 phosphosites in other proteins that exhibit the highest frequency of positive and negative co-regulation with the (**A**) S853, (**B**) S1212, and (**C**) S1239 predominant phosphorylation sites of KIF21A.

To characterize the phospho-signaling dynamics associated with the predominant KIF21A phosphosites, we performed a systematic co-regulation analysis. This analysis aimed to identify the phosphosites in other proteins (PsOPs) that consistently exhibited coordinated regulation with the predominant KIF21A phosphosites across multiple independent phosphoproteomics datasets. Predefined criteria were applied to define significant co-regulation, including Fisher’s exact test (FET) *p*-value < 0.05, a minimum frequency threshold of ≥10% relative to the total differential frequency of the predominant phosphosite, support from at least three independent publications (PMID confidence) and reproducible regulation across a minimum of three distinct experimental conditions, as assessed by code count. Following application of these criteria, KIF21A (S853) showed positive co-regulation with 1075 PsOPs and negative co-regulation with 156 PsOPs (Supplementary Tables S3 and S4). Similarly, S1212 exhibited positive and negative co-regulation with 428 and 20 PsOPs, respectively, whereas S1239 displayed positive co-regulation with 1016 PsOPs and negative co-regulation with 131 PsOPs (Supplementary Tables S5–S8). A comparative analysis was performed to identify PsOPs commonly co-regulated with the predominant phosphorylation sites of KIF21A. Analysis of positively co-regulated PsOPs revealed that 91 PsOPs were commonly shared between S853 and S1236, whereas 50 and 16 PsOPs were shared between S1212 and S1239, and between S1212 and S853, respectively. Likewise, analysis of negatively co-regulated PsOPs revealed that four PsOPs were shared between S853 and S1236, whereas one PsOP was shared between S1212 and S853 (Supplementary Figure S2). Figure 3 illustrates the co-regulation patterns of the top co-regulated proteins, indicating these patterns are consistent across the datasets. For KIF21A (S853), Nuclear cap-binding protein subunit 1 (NCBP1) (S22), Sodium/hydrogen exchanger 1 (SLC9A1) (S703), and RalBP1-associated Eps domain-containing protein 1 (REPS1) (S709) are the most positively co-regulated PsOPs, with frequencies of 30, 27, and 26 occurrences across independent datasets, respectively. In contrast, Ankyrin-3 (ANK3) (S4350), Apoptotic chromatin condensation inducer in the nucleus (ACIN1) (S328), and Integrin alpha-4 (ITGA4) (S1021) represented the most prominent negatively co-regulated PsOPs, with frequencies of 17,14 and 12 occurrences, respectively (Figure 3A). For KIF21A (S1212), the highest positive co-regulation was observed with Telomeric repeat-binding factor 2 (TERF2) (S365), Ubiquitin recognition factor in ER-associated degradation protein 1 (UFD1) (S247), and Centro-somal protein of 170 kDa (CEP170) (S1529), detected with frequencies of 52, 40, and 38, respectively. Conversely, Protein DEK (DEK) (S243), Nucleolar MIF4G domain-containing protein 1 (NOM1) (S317), and Protein DEK (DEK) (S244) constituted the most frequently negatively co-regulated PsOPs, with frequencies of 24, 23, and 23, respectively (Figure 3B). Similarly, KIF21A S1239 showed strong positive co-regulation with Leucine-rich repeat and WD repeat-containing protein 1 (LRWD1) (S243), Serine/threonine-protein kinase PAK 2 PAK2 (S141), and F-actin-uncapping protein LRRC16A (CARMIL1) (S1094), each detected with frequencies of 26, 26, and 24 datasets, respectively. In contrast, Serine/threonine-protein kinase PRP4 homolog (PRP4K) (S294), Transformer-2 protein homolog alpha (TRA2A) (S2), and Serine/threonine-protein kinase PRP4 homolog (PRP4K) (S368) were identified as the most prominent negatively co-regulated PsOPs for this site (Figure 3C).

### 2.4. Phosphosite-Specific Biological Functions of Co-Regulated PsOPs

**Figure 4:**
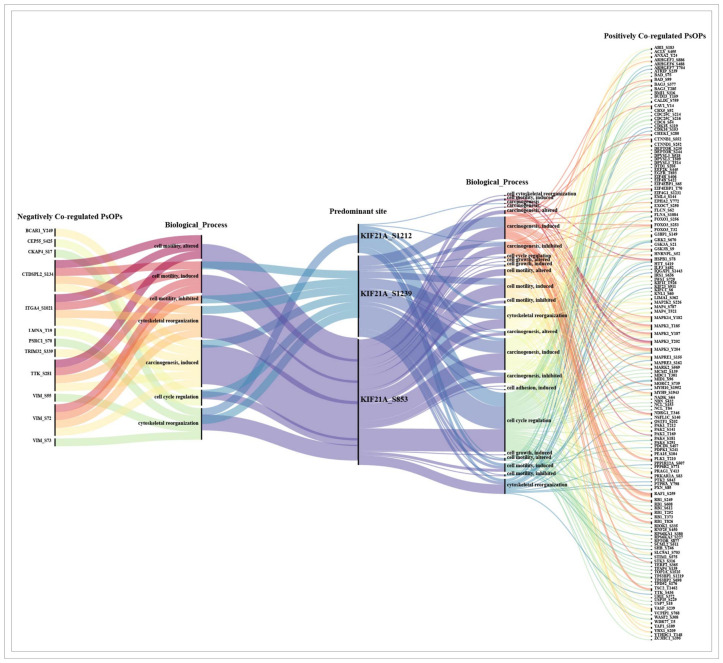
Association of predominant KIF21A phosphosites (S1212, S853, S1239) with positively and negatively co-regulated PsOPs and their related biological processes. The predominant KIF21A phosphosites associated with PsOPs involved in carcinogenesis, cytoskeletal organization, cell cycle regulation, and cell motility.

In recent years, an increasing number of studies have focused on KIF21A to elucidate its cellular functions and involvement in various diseases [18,19,20]. Although multiple phosphosites in KIF21A have been identified, the biological functions regulated by these sites are yet to be characterized. To address this, we employed a “guilt-by-association” strategy to infer phosphosite-specific functions of KIF21A by analyzing the biological roles of co-regulated PsOPs. Co-regulation analysis enables the identification of functional relationships between proteins that do not physically interact or colocalise [21]. The phosphosite-specific biological functions of co-regulated PsOPs were obtained from the PhosphoSitePlus database. The PsOPs co-regulated with the KIF21A predominant phosphosite were mainly involved in carcinogenesis, cytoskeletal organization, cell cycle regulation, and cell motility (Figure 4).

### 2.5. Functional Enrichment of Positively and Negatively Co-Regulated PsOPs

**Figure 5:**
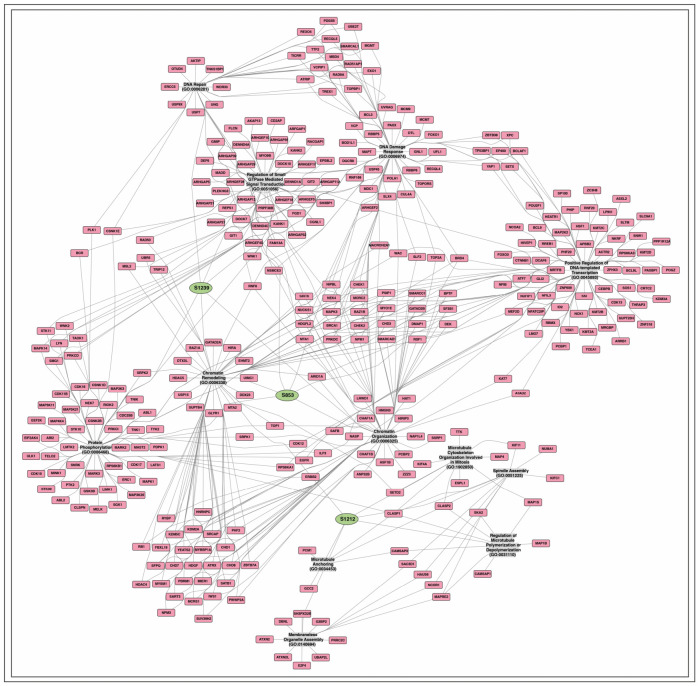
Representation of biological processes associated with positively co-regulated phosphoproteins specific to KIF21A predominant phosphosites (S853, S1212, and S1239).

To investigate the biological relevance of the positively co-regulated proteins associated with the predominant KIF21A phosphosites, a functional enrichment analysis was performed using Enrichr [22]. Proteins positively co-regulated with KIF21A (S853, S1239) were significantly enriched in biological processes related to chromatin remodeling, chromatin organization, and DNA damage response. In contrast, proteins co-regulated with KIF21A (S1212) were predominantly enriched in processes associated with microtubule polymerisation/depolymerisation, microtubule anchoring, and cytoskeleton organization, consistent with the established role of KIF21A in microtubule dynamics (Figure 5).

**Figure 6:**
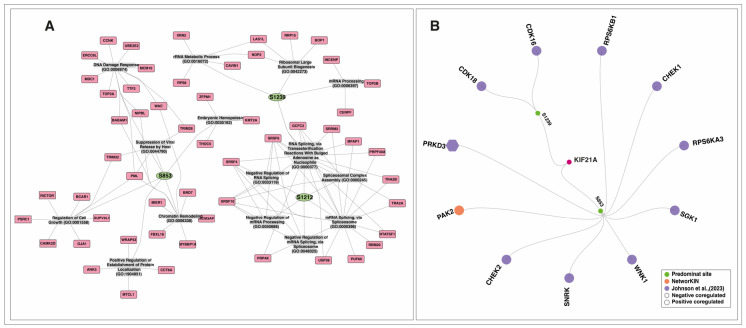
(**A**) The network diagram depicts the biological processes associated with negatively co-regulated phosphoproteins specific to KIF21A predominant phosphosites (S853, S1212, and S1239). (**B**) Circular dendrogram representing upstream regulators associated with KIF21A phosphorylation sites. This network includes both predicted kinases and kinases reported by Johnson et al. (2023) [23], which show phosphorylation co-regulation with the corresponding KIF21A phosphosites.

Proteins negatively co-regulated with KIF21A (S853) were significantly enriched in biological processes related to DNA damage response, embryonic hemopoiesis, and regulation of the establishment of protein localization. Similarly, proteins negatively co-regulated with KIF21A (S1212) were predominantly enriched in processes associated with mRNA Splicing, mRNA processing, and spliceosome-related functions. In addition, proteins negatively co-regulated with KIF21A (S1239) were enriched in mRNA splicing via the spliceosome, RNA splicing via transesterification reactions with bulged adenosine as a nucleophile, and mRNA processing (Figure 6A).

### 2.6. Site-Specific Upstream Kinase Co-Regulation Associated with Predominant KIF21A Phosphosites

Analysis of upstream regulators revealed distinct co-regulation patterns with the predominant KIF21A phosphosites. For the S853 predominant phosphosite, p21 (RAC1) activated kinase 2 (PAK2) (S152, S141) was identified as a predicted upstream regulator among positively co-regulated phosphosites using the kinase prediction tool, NetworKIN. In addition, the upstream regulators of KIF21A (S853), namely Ribosomal protein S6 kinase alpha-1 RPS6KA1 (S380, S732, S363, and S369), Ribosomal protein S6 kinase beta-1 RPS6KB1 (S427), Serine/threonine-protein kinase Chk1 CHEK1 (S280), Ribosomal protein S6 kinase alpha-3 RPS6KA3 (S227, S375), Serine/threonine-protein kinase SGK1 (S401), Serine/threonine-protein kinase WNK1 (S167), SNF-related serine/threonine-protein kinase SNRK (S569), and Serine/threonine-protein kinase Chk2 CHEK2 (S260) showed positive co-regulation and PRKD3 (S216) showed negative co-regulation were extracted from Johnson et al. (2023) [23]. For S1239, upstream kinases CDK18 (S132 and S74) and CDK16 (S95 and S138) were extracted from Johnson et al. (2023) [23] and identified among positively co-regulated PsOPs. In contrast, no upstream kinases or predictive kinases were co-regulated with S1212 (Figure 6B, Supplementary Table S9).

### 2.7. Cancer-Specific Correlation Between the KIF21A S1239 and Phosphosites in Predicted Upstream Regulators and Carcinogenesis-Associated PsOPs

Using cProSite-based correlation analysis across multiple tumor types, we examined the relationship between phosphorylation of the predominant KIF21A site and phosphorylation of candidate upstream kinases and regulatory proteins identified by Johnson et al. (2023) [23]. Cancer-specific expression analysis revealed site-specific correlations between KIF21A phosphorylation and upstream kinases. Across multiple cancer types, including uterine, lung, kidney, and brain cancers, the KIF21A S1239 phosphorylation site exhibited moderate-to-strong positive correlations with CDK18 phosphorylation at S74 and S132 (Supplementary Figure S1).

**Figure 7:**
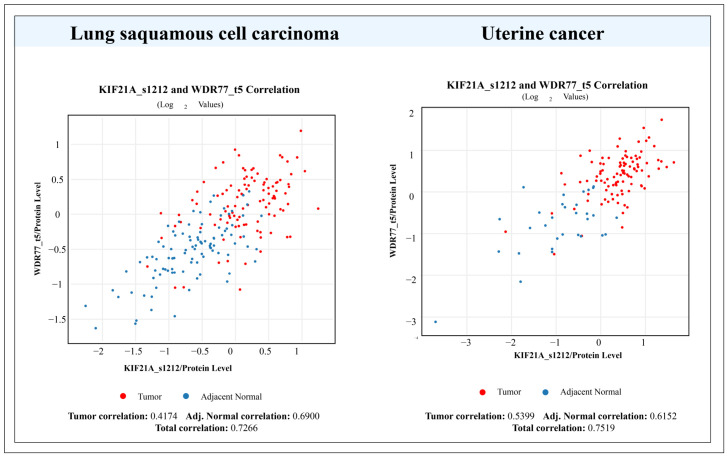
Scatter plot showing the correlation between KIF21A S1212 and WDR77 T5 phosphosite levels in lung squamous cell carcinoma and uterine cancer.

Additionally, correlation analysis was performed between KIF21A S1239 and S1212 and their co-regulated phosphosites associated with carcinogenesis using cProSite. Our analysis identified a positive phosphorylation co-regulation of PAK2 (S141) with KIF21A (S1239) [24] (Supplementary Figure S1). In contrast, for KIF21A (S1212) among the co-regulated proteins, WDR77 (T5) exhibited significant expression correlations across multiple cancer types, including brain cancer, kidney cancer, lung squamous cell carcinoma, and uterine carcinoma. Notably, although several additional co-regulated PsOPs may have roles in cancer, their phosphorylation data were not represented in cProSite [25,26,27] (Figure 7). Notably, cProSite data showed comparatively higher correlation values in tumor samples than in adjacent normal tissues across several cancer types. Since cancer-associated phosphoproteomic data for KIF21A S853 were not available in cProsite, this site was not included in the cProsite-based cancer correlation analysis presented in this section.

### 2.8. Binary and Complex Interactors Associated with KIF21A Predominant Phosphosites

**Figure 8:**
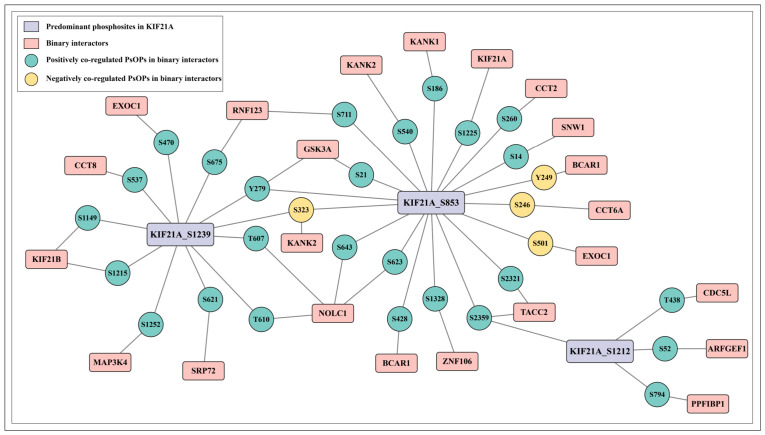
Protein–protein interaction network of KIF21A and its binary interactors. The interaction network illustrates the positive and negative co-regulation of KIF21A predominant phosphosites with phosphosites in binary interactors.

**Figure 9:**
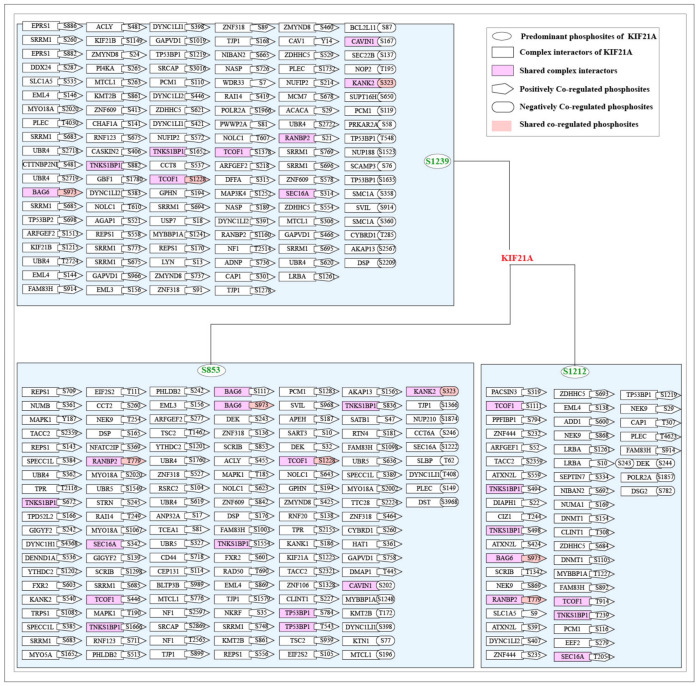
Protein–protein interaction network of KIF21A complex interactors, showing positively and negatively co-regulated phosphosites (PsOPs). Nodes represent complex-associated phosphosites. Pink indicates shared interactors across KIF21A phosphosites, while peach denotes phosphosites commonly co-regulated with multiple KIF21A phosphosites.

Protein–protein interaction analysis further demonstrated extensive signaling connectivity among the proteins co-regulated with predominant phosphosites in KIF21A. For (S853), 14 binary interactors and 114 complex interactors were observed in positively co-regulated PsOPs, while four binary and 15 complex interactors were identified in negatively co-regulated PsOPs. In the case of S1212, four binary and 45 complex interactors were positively co-regulated, whereas four complex interactors were negatively co-regulated. For S1239, 10 binary interactors were positively co-regulated and one binary interactor was negatively co-regulated; additionally, 99 complex interactors showed positive co-regulation and 18 showed negative co-regulation (Figure 8 and Figure 9, Supplementary Tables S10 and S11).

### 2.9. Phosphosite-Specific Interaction and Kinase Network Analysis of KIF21A Within Cortical Microtubule Stabilization Complexes

Cortical microtubule stabilization complexes (CMSCs) are specialized protein assemblies located at the cell cortex that capture and stabilize the plus ends of microtubules [28]. These complexes are composed of KANK family proteins (KANK1 and KANK2), Liprin family adaptors (PPFIA1 and PPFIBP1), CLASPs (CLASP1 and CLASP2), ERC1, PHLDB2, and KIF21A, forming an integrated platform that connects microtubule dynamics to cortical adhesion sites [29]. KIF21A functions as an anterograde ATP-dependent motor protein and has been shown to interact in vitro with the KN motif and ankyrin repeat domain-containing protein 1 (KANK1), a regulator of actin polymerization. At the cell cortex, KIF21A associates with KANK1-Pleckstrin homology-like domain family B member 2 LL5B (PHLDB2) complex, which contributes to the stabilization of microtubule dynamics [12,13,30,31].

In our phosphoproteomic analysis, we observed site-specific co-regulation of KIF21A phosphosites in distinct components of the CMSCs. Interestingly, KIF21A predominant phosphosite S853 exhibited strong positive co-regulation with phosphosites in canonical CMSC members, including KANK1 (S186); ERC1 (S37); PHDLB2 (S513, S42); and CLASP1 (S600, S572, and S646). In contrast, KIF21A (S1212) displayed co-regulation with a partially overlapping but distinct subset of CMSC-associated phosphosites, PPFIA1 (S708 and S1172), PPFIBP1 (S794), CLASP2 (S952), and CLASP1 (S1091). Similarly, KIF21A (S1239) showed co-regulation with ERC1 (S21, S17), PPFIA1 (S242), CLASP1 (S1091), and CLASP2 (S455 and S8). This co-regulation suggests that site-specific phosphorylation of KIF21A may preferentially influence its involvement in cortical microtubule regulatory pathways, although further experimental validation will be required to establish a direct mechanistic role.

Furthermore, we evaluated whether the upstream kinases identified for KIF21A are capable of phosphorylating other proteins within the CMSCs. To address this, experimentally validated and computationally predicted substrates of KIF21A-associated upstream kinases (mentioned in Figure 5) were compiled from databases such as PhosphoSitePlus, Phospho.ELM 9.0, and RegPhos 2.0. In addition, predicted substrates were incorporated from kinase-substrate prediction tools and previously reported datasets (Johnson et al., 2023; Sugiyama et al.,2019) [23,32]. Intersection analysis was then performed between these downstream substrates and the proteins within the CMSCs. The analysis revealed that the upstream kinases of KIF21A, including CHEK2, RPS6KA1, RPS6KA3, RPS6KB1, SGK1, WNK1, and PAK2, were associated with phosphorylation of one of the CMSC proteins, KANK1 (S186), based on substrate data derived from Johnson et al. (2023) [23] and the kinase-substrate prediction tool. This suggests a potential shared kinase regulatory axis, in which KIF21A-associated upstream kinases may coordinately regulate phosphorylation events in CMSCs.

### 2.10. Pan-Cancer Gene Expression Profiling of KIF21A

**Figure 10:**
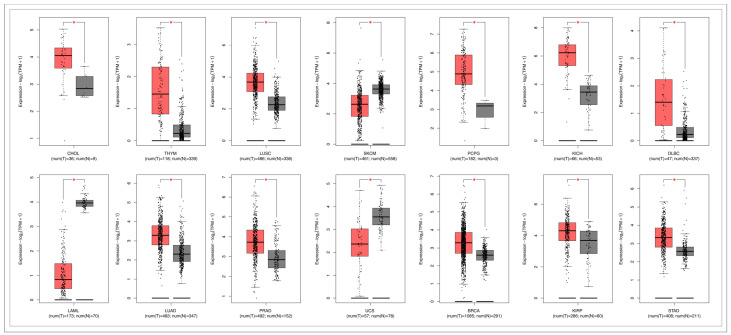
Differential gene expression analysis of KIF21A across multiple cancer types. Box plots show the distribution of KIF21A gene expression in tumor (red) and normal (gray) samples for each cancer type, * indicates statistical significance.

The gene-level expression pattern of KIF21A across multiple cancer types was analyzed using the GEPIA2 database. A log2fold change (FC) of 0.58 and a *p*-value < 0.025 was applied to identify significant differential expression of KIF21A in cancer samples compared to normal samples. A total of 14 cancer types showed significant alterations in KIF21A expression. Among these 11 cancer types, such as breast invasive carcinoma (BRCA), cholangio carcinoma (CHOL), lymphoid neoplasm diffuse large B-cell lymphoma (DLBC), kidney chromophobe (KICH), kidney renal clear cell carcinoma (KIRP), lung adenocarcinoma (LUAD), lung squamous cell carcinoma (LUSC), pheochromocytoma and paraganglioma (PCPG), prostate adenocarcinoma (PRAD), stomach adenocarcinoma (STAD), and thymoma (THYM), exhibited upregulation of KIF21A, whereas three cancer types, including acute myeloid leukemia (LAML), uterine carcinosarcoma (UCS), and skin cutaneous melanoma (SKCM), showed downregulation (Figure 10).

**Figure 11:**
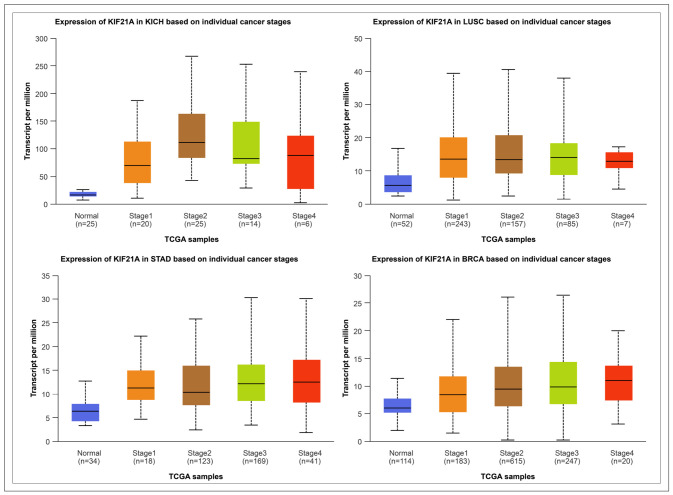
Stage-wise expression analysis of KIF21A across multiple cancer types from TCGA datasets. Box plots display normal tissue and tumor samples stratified by clinical stages (stage 1 to stage 4).

Further, stage-wise expression analysis of KIF21A across 14 cancer types was performed using the UAL-CAN database to evaluate its clinical significance. The analysis revealed progressively increased KIF21A expression across cancer stages, particularly in BRCA, STAD, and LUSC, suggesting a potential role for KIF21A in cancer progression (Figure 11).

### 2.11. Gene Expression Correlation Analysis of Proteins Co-Regulated with KIF21A

Since phosphorylation may correlate with parent protein abundance, we examined whether the corresponding proteins exhibit coordinated expression patterns at the transcript level using the UALCAN database. A comparative analysis was performed by intersecting the positively and negatively co-regulated proteins of KIF21A with genes positively and negatively co-regulated with KIF21A across 14 cancer datasets. Proteins co-regulated with each of the three predominant phosphosites were evaluated independently. The results indicated that positively associated proteins exhibited consistent correlation with KIF21A at the gene level across cancers, whereas minimal correlation was observed for negatively associated proteins (Supplementary Tables S12 and S13).

Among the positively co-regulated phosphoproteins, those associated with KIF21A (S853) exhibited the highest concordance with gene-level co-regulation across cancer types, followed by KIF21A (S1239) and KIF21A (S1212). Among the observed correlations, the highest Pearson correlation coefficients were observed for PTK2 (0.77) and CTNND1 (0.74) in thymoma (THYM). Interestingly, many of the phosphoproteins involved in carcinogenesis and cytoskeletal organization exhibited concordant gene-level co-regulation with KIF21A across almost all cancer types. For instance, it includes PTK2, CTNND1, YAP1, CHEK1, MAPK1, FOXO3, CTNNB1, and RPS6KA3, among others (Figure 4). Among the negatively co-regulated phosphoproteins, only those co-regulated with the KIF21A (S853) site showed a detectable gene-level association only in THYM.

Further, we examined whether any of the upstream kinases were present among these correlated genes and found positive gene-level correlation between RPS6KA3 and KIF21A in cancers such as KICH, LUSC, CHOL, KIRP, and THYM. In addition, upstream kinases such as CHEK1, PAK2, and CDK16 also showed gene-level correlation across different cancer types. Moreover, among the components of CMSCs, KANK1 and ERC1 showed positive gene-level correlations with KIF21A across multiple cancers, including SKGM, UCS, KIRP, LUAD, and THYM (Supplementary Tables S12 and S13).

## 3. Discussion

KIF21A is a microtubule-dependent kinesin-4 motor protein that plays an essential role in anterograde transport and regulation of cortical microtubule dynamics, particularly in neuronal contexts [19,33]. KIF21A has been reported to interact with the guanine nucleotide-exchange factor BIG1, which maintains Golgi organization, and its depletion may impair lysosomal trafficking and function [34]. Genetic studies have demonstrated that point mutations in KIF21A can cause congenital fibrosis of the extraocular muscles type 1 (CFEOM1) [11]. It also plays an important role in various cancer types, such as breast cancer [19,35], pulmonary adenocarcinoma [36], and pancreatic ductal adenocarcinoma [37].

In our phosphoproteomic analysis, we identified three predominant phosphosites in KIF21A, among which S853 showed a distinct enrichment toward microtubule stabilization pathways. In addition, KIF21A (S853) exhibited preferential co-regulation with components of the cortical microtubule stabilization complex, KANK1 (S186)-PHLDB2 (S513, S42), which is known to mediate microtubule stabilization at the cell cortex [30]. S853 coregulates with CMSCs, including KANK1 (S186), PHLDB2 (S513, S42), and CLASP1 (S1091) sites, reinforcing links to KANK1 (S186)-PHLDB2 (S513, S42) for plus end stabilization. This phosphorylation pattern suggests a potential association of KIF21A (S853) with cortical microtubule stabilization complexes, and possible involvement in microtubule growth-related processes.

Cancer-specific correlation of upstream kinases with three predominant phosphosites was analyzed using the cProSite [38]. Among the three predominant phosphosites, only KIF21A (S1239) showed tumor-associated correlations, particularly with CDK18 (S74 and S132), consistent with findings from Johnson et al. (2023) [23]. Dysregulation of CDK18 has been reported in many diseases, including metabolic disorders, cerebral ischemia, depression, cancer, and Alzheimer’s disease [39]. The observed correlation between KIF21A (S1239) and CDK18 phosphorylation may indicate a possible association with cancer-relevant kinase networks. However, this finding warrants further experimental validation.

Further, correlation analysis was performed between predominant KIF21A phosphosites and their carcinogenesis-associated co-regulated proteins. KIF21A (S1239) showed a positive correlation with PAK2 (S141) in lung squamous cell carcinoma, whereas KIF21A (S1239) was correlated with WDR77 (T5) in multiple cancers. PAK2 is associated with the development of cancers such as lung squamous cell carcinoma, ovarian cancer, and pancreatic cancer [40]. Activation of PAK2 promotes cancer progression by inducing enhanced cell proliferation, survival, invasion, and metastasis [41]. WDR77 is a WD-40 repeat-containing protein and is an important regulator of cellular pathways involved in cancer progression [42]. Overexpression of WDR77 promotes cell proliferation and suppresses immune-related pathways in various cancers, including colorectal, lung, breast, and stomach cancer, among others [43]. WDR77 was also identified as a prognostic marker in gliomas and ovarian cancer [44,45]. These findings indicate that phosphosites such as S1212 and S1239 are associated with tumor context-dependent signaling patterns involving carcinogenesis-related proteins.

Pan-cancer expression analysis indicates that KIF21A was broadly dysregulated across multiple tumor types, with the predominant upregulation observed in cancers such as BRCA, LUAD, LUSC, PRAD, and THYM. Elevated KIF21A expression has been associated with poor prognosis in early-stage pancreatic ductal adenocarcinoma (PDAC) patients following pancreaticoduodenectomy, suggesting its potential utility as a prognostic biomarker [38]. The consistent increase in expression along with its stage-wise elevation in cancers suggests that KIF21A may contribute to tumor progression rather than merely reflecting passive expression changes. Given its established role in microtubule dynamics and intracellular transport, elevated KIF21A levels may support processes such as cytoskeletal remodeling, which are critical for cancer cell invasion and metastasis [46,47].

At the gene expression level, the observed asymmetry between positively and negatively co-regulated PsOPs suggests that phosphorylation-driven signaling relationships are selectively reflected at the transcriptional level. The strong gene-level correlations observed for PTK2 (S840 and S843) at KIF21A (S853) and CTNND1 (S320) at KIF21A (S1239) phosphosite, particularly in THYM. PTK2 (S840 and S843) gene expression has been associated with the pathological stage, progression, and cancer cell survival [48]. Dysregulated expression of CTNND1 has been observed in gastric cancer, hepatocellular carcinoma, and breast cancer and is associated with cancer metastasis [49,50,51]. Similarly, CTNND1 participates in cell signaling, transcriptional regulation, and cytoskeletal organization [50], and its reduced expression has been observed in colon, bladder, and breast cancers and is associated with poor prognosis [49]. The strong correlation observed for CTNND1 further supports the involvement of KIF21A in cytoskeleton-associated signaling networks in cancer. The observed gene-level correlation of upstream kinases such as RPS6KA3, CHEK1, PAK2, and CDK16 may indicate potential associations of KIF21A within a kinase-driven regulatory network. The observed positive gene-level correlations of CMSCs, such as KANK1 and ERC1 with KIF21A across multiple cancer types, suggest that KIF21A may be part of a broader regulatory network involved in cytoskeletal remodeling in cancer.

The data-driven findings from this study highlight the importance of site-specific phosphorylation in regulating KIF21A function across different biological contexts. The observed variability in phosphosite-associated correlations and kinase interaction suggests that KIFF21A may operate through distinct regulatory mechanisms depending on cellular and disease conditions. The identification of conserved and cancer-associated phosphosites supports the idea that specific residues may serve as critical regulatory nodes within broader signaling networks. These insights provide a framework for exploring how phosphorylation may fine-tune KIF21A activity and its integration into cytoskeletal and signaling pathways. Furthermore, given the limited phosphoproteomic characterization available for KIF21A, this study provides an initial framework to systematically explore its phosphosite level regulation. By integrating large-scale datasets, we highlight potential links between KIF21A phosphorylation and disease-associated signaling, particularly in cancer. These findings serve as hypothesis-generating insights that can guide future experimental studies to validate and expand upon the predicted regulatory mechanisms. In this context, targeted investigations may further clarify the biological significance of KIF21A phosphosites and their potential relevance in disease progression and therapeutic intervention.

## 4. Materials and Methods

### 4.1. Assembly and Curation of Global Phosphoproteomics Datasets for KIF21A

To systematically characterize phosphosite-specific regulation associated with KIF21A, we assembled and curated publicly available human cellular phosphoproteomics studies through a comprehensive literature survey. The assembly and analysis of the global phosphoproteomic datasets of KIF21A employed the previously established methodology developed by our team [52,53,54]. A PubMed search was performed using the query terms “phosphoproteomics” OR “phosphoproteome” while excluding “Plant” and “Review” articles. From the resulting literature, we identified studies reporting high-throughput human phosphoproteome data that included KIF21A phosphosites and were subjected to data curation. These datasets are classified as qualitative profile datasets (test conditions and control as independent phosphosite profile datasets) and quantitative differential datasets (directly compared test biological or experimental conditions against their corresponding controls).

The curated datasets originated from multiple experimental conditions and utilized a range of mass spectrometry-based data acquisition workflows, including labeled and label-free quantification strategies. To ensure high confidence phosphosite identification and limit potential false positives, we focused exclusively on phosphosites with high localisation confidence, defined as a localisation probability of ≥75% or an A-score > 13, as higher values reflect increased confidence in site assignment. To further enhance biological relevance and statistical robustness, differential datasets were filtered using a significance threshold of *p* < 0.05, along with fold-change cutoffs of ≥1.3 for upregulated sites and ≤0.76 for downregulated sites [55,56].

Phosphorylation datasets were additionally grouped based on the residue type enriched in each study (Ser/Thr/Tyr). Individual phosphosites were mapped to their corresponding UniProt accessions using an in-house phosphosite mapping pipeline (UniProt release on 13 May 2023) [29], followed by gene symbol standardization based on HGNC annotations [57]. The curated and harmonized datasets were subsequently used for phosphosite frequency analysis, differential regulation assessment, and downstream co-regulation and interaction analyses.

### 4.2. Identification of Predominant Phosphosites in KIF21A

To identify the predominant phosphosites of KIF21A, Human cellular global phosphoproteomic datasets enriched for Serine (S), Threonine (T), and Tyrosine (Y) residues were systematically analyzed. All confidently localized KIF21A phosphosites were extracted from the curated qualitative profile and quantitative differential phosphoproteomic datasets. Each phosphorylation site was ranked according to its frequency of detection across the assembled datasets, enabling prioritization of consistently observed phosphorylation events. For quantitative datasets, phosphosites exhibiting statistically significant differential regulation were additionally considered. Phosphosites S853, S1212, S1239 represent 50% of KIF21A phosphorylation events. To visualize the distribution and frequency of KIF21A phosphosites along the protein sequence, lollipop plots were generated using the R/Bioconductor package trackViewer [58] to visualize the phosphorylation sites representing KIF21A.

### 4.3. Identification of Similarly and Oppositely Co-Regulated Phosphosites Predominant to KIF21A Phosphosites

To investigate phosphorylation site-specific co-regulation associated with KIF21A, quantitative human cellular phosphoproteomics datasets were further analyzed to identify phosphosites in other proteins (PsOPs) that exhibited coordinated regulation with the predominant KIF21A phosphosites (S853, S1212, and S1239). Analyses were performed independently for each predominant KIF21A phosphosite to ensure site-specific resolution. Given the heterogeneity of experimental conditions, biological systems, and analytical platforms across studies, raw data re-analysis was not feasible. Therefore, curated quantitative differential datasets were used irrespective of potential overrepresentation of specific experimental contexts. For each predominant KIF21A phosphosite, datasets were categorized based on the regulation status of the site as either upregulated (U) or downregulated (D). Within each category, PsOPs were classified according to their corresponding regulation status, resulting in four regulatory combinations: UU, UD, DU, and DD. PsOPs that were upregulated or downregulated concordantly with KIF21A phosphosites (UU and DD) were considered candidates for positive co-regulation, whereas PsOPs showing opposing regulation (UD and DU) were considered candidates for negative co-regulation. To quantify co-regulatory relationships, PsOPs of KIF21A phosphosite pairs were grouped into UUDD (positive co-regulation) and UDDU (negative co-regulation) categories.

A one-sided Fisher’s exact test (FET) was applied using contingency tables that captured the co-occurrence of differential regulation across all quantitative datasets [59,60,61,62]. PsOP pairs exhibiting a *p*-value < 0.05 in either the UUDD or UDDU category were considered statistically significant.

$$\sum p=\frac{\left(a+b\right)!\left(c+d\right)!\left(a+c\right)!\left(b+d\right)!}{n!}\sum_{i}\frac{1}{a_{i}!b_{i}!c_{i}!d_{i}!}$$

Here the components of the contingency table were defined as follows: “a” n_00 represents the number of experimental conditions in which both phosphosites were not detected. “b” (n_U0 + n_D0 + n_0U + n_0D) denotes the number of conditions where one phosphosite was regulated (either upregulated or downregulated) while the other site was not detected. “c” (n_UD + n_DU) corresponds to the number of experimental conditions showing opposite co-regulation between two phosphosites. “d” (nUU + nDD) represents the number of conditions in which both phosphosites exhibited identical co-regulation (both were either upregulated or downregulated).

To minimize bias arising from repeated experimental conditions, overrepresentation of specific stimuli, or disproportionate reporting from individual studies, additional filtering criteria were applied. Significant PsOPs pairs were further evaluated using the ratio of concordant to discordant regulation events, with a minimum threshold corresponding to at least 10% of the total number of datasets in which the respective KIF21A phosphosite was identified. Furthermore, to ensure biological robustness, PsOPs of KIF21A phosphosite pairs were required to be observed in at least three independent studies (distinct PubMed IDs) and experimental conditions. This multi-step filtering method reduced bias related to the dataset and ensured that the remaining phosphosite associations reflected reproducible and biologically meaningful co-regulatory patterns. The high-confidence positively and negatively co-regulated PsOPs identified for each predominant KIF21A phosphosites were subsequently used for downstream analyses, including functional enrichment, protein–protein interaction analysis, and kinase-substrate relationship inference.

### 4.4. Identification of KIF21A Protein–Protein Interactions

To systematically characterize the interaction network of KIF21A, both experimentally validated and predicted protein interactors were compiled from multiple curated protein–protein interaction databases. Binary and complex interactors of KIF21A were retrieved from the Human Protein Reference Database (HPRD) [63], Biomolecular Interaction Network Database (BIND) [64], BioGRID [65], ConsensusPathDB release 35 [66], NetPath [67], CORUM [68], and RegPhos 2.0 [69]. These datasets were integrated to construct a comprehensive interaction map of KIF21A-associated proteins.

### 4.5. Identification of Upstream Kinases of KIF21A

To investigate the upstream signaling landscape of KIF21A, potential kinases targeting its phosphosites were systematically identified. Kinase prediction and annotation were performed using complementary computational and experimental resources. Kinases associated with specific KIF21A phosphosites were retrieved from NetworKIN (accessed on 4 January 2023) [70] and AKID (accessed on 24 May 2023) [71]. Experimentally derived kinase-substrate relationships were obtained from iKiP-DB [72], PhosphoSitePlus (downloaded on 22 May 2023) [7]. In addition, kinase associations reported by Johnson et al. (2023) based on synthetic peptide screening across the human kinome were incorporated, using a 90th percentile confidence cutoff [23].

### 4.6. cProSite-Based Cancer Phosphoproteomic Analysis

The Cancer Proteogenomic Data Analysis site (cProSite) [38] is an interactive web-based resource designed for the exploration and visualization of large-scale proteomic and phosphoproteomics datasets. To assess the correlation of KIF21A phosphosites with cancer-associated phosphorylation patterns, cProSite (https://cprosite.ccr.cancer.gov/, access date: 9 November 2023) was utilized.

### 4.7. Functional Enrichment Analysis and Data Visualization

The functional gene enrichment analysis of positively and negatively co-regulated proteins with KIF21A was analyzed by using the Enrichr tool [22]. Multiple sequence alignment (MSA) was performed using Clustal Omega (v1.2.4) (https://www.ebi.ac.uk/jdispatcher/msa/clustalo, access date: 12 April 2026) [73]. Network construction and visualization were carried out using Cytoscape v3.10.4 [74], PathVisio3 [75], and Adobe Illustrator (2020). RAWGraph 2.0 was used for generating a dendrogram (https://www.rawgraphs.io/, access date: 12 April 2026).

### 4.8. Gene Expression Analysis of KIF21A

Gene-level expression of KIF21A across 31 human cancer types was evaluated using the Gene Expression Profiling Interactive Analysis (GEPIA2, version 2) [76] web server, which integrates RNA sequencing data from The Cancer Genome Atlas (TCGA) and the Genotype–Tissue Expression (GTEx) projects. A threshold of log2 fold change ≥ 0.58, and a significance cutoff of *p* < 0.05 were used to identify statistically significant alterations in KIF21A gene expression.

To investigate the variation in KIF21A expression across different stages of cancer progression, stage-wise expression analysis was conducted using TCGA patient data available through the University of ALabama at Birmingham CANcer (UALCAN) [77] data analysis portal. Statistical comparisons among multiple groups were performed using one-way analysis of variance (ANOVA), and a Pr (>F) value < 0.05 was considered statistically significant.

### 4.9. Gene-Level Expression Correlation Between KIF21A and Co-Regulated Proteins

We further analyzed the gene expression correlation between KIF21A and co-regulated proteins using gene co-expression data for KIF21A across individual cancer types, retrieved from the UALCAN database.

Genes exhibiting positive and negative co-regulation with KIF21A extracted from UALCAN were subsequently intersected with the positively and negatively co-regulated proteins of KIF21A, respectively.

## 5. Conclusions

In this study, we performed a comprehensive integrative phosphoproteomic analysis to investigate site-specific regulatory mechanisms associated with KIF21A, a kinesin-4 motor protein involved in microtubule dynamics and neuronal function. Our analysis suggests that different phosphorylation sites on KIF21A may be linked to different cellular processes, such as cytoskeletal organization, intracellular transport, and regulation through autoinhibition, indicating that its regulation is likely context-dependent rather than uniform. These findings provide a broader view of how KIF21A could participate in multiple cellular pathways through site-specific regulation. Further studies are needed to experimentally validate these observations and to understand how individual phosphorylation sites may affect KIF21A function. Approaches such as mutational analysis and functional assays will help clarify their roles in cellular processes and disease conditions.

## 6. Limitations and Future Directions

Although this study presents a site-specific phosphoproteomic analysis of KIF21A, certain limitations must be considered, as the findings are derived from an integrative analysis of publicly available phosphoproteomics datasets that inherently vary in experimental design, sample preparation, and quantification strategies. Despite applying filtering criteria to ensure high confidence co-regulation, inter-study variability may influence phosphosite detection and regulatory patterns. The identified correlation relationships and kinase-substrate associations are derived from statistical and database-based analyses, and direct experimental validation was not performed. Therefore, the functional significance of key phosphosites, particularly KIF21A (S853) in cortical microtubule regulation and KIF21A (S1239) in tumor-associated signaling, remains to be experimentally confirmed. Correlation with cancer database does not establish causality, and further studies using phospho-mutagenesis and cellular functional analyses, in vitro kinase assays, phosphosite-specific antibody, microtubule binding assays, kinase co-expression/phosphorylation assessments, phosphorylation-deficient and phosphomimetic mutants will be necessary to validate these findings. We analyzed the gene-level correlation of co-regulated phosphoproteins with KIF21A; however, protein-level expression validation is required to better understand the relationship between parent protein abundance and phosphosite-specific regulation. Nevertheless, this work provides a structured framework for future investigations into site-specific regulation of KIF21A and its role in cytoskeletal and proliferative signaling pathways.

## Data Availability

The datasets supporting the conclusions of this article are included within the article and its Supplementary Files.

## Supplementary Materials

The following supporting information can be downloaded at: https://www.mdpi.com/article/10.3390/ijms27146387/s1.
