# Supplementary figures and images for: Deciphering Site-Specific Regulatory Networks of the Kinesin Protein KIF21A Through Integrative Phosphoproteomic Analysis
Source: Int J Mol Sci. 2026 Jul 18;27(14):6387. doi: 10.3390/ijms27146387 (PMC13411604; doi:10.3390/ijms27146387)

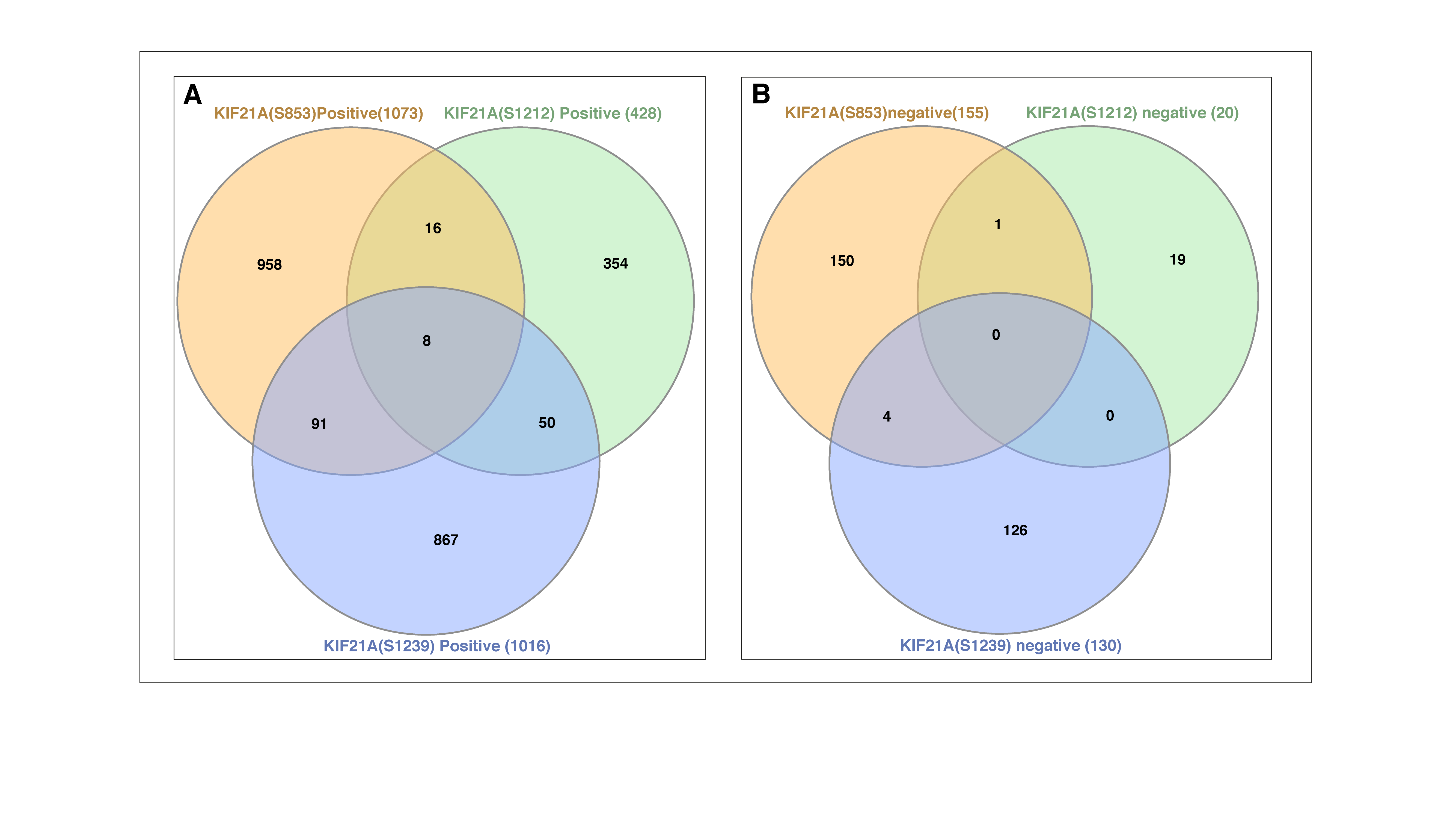

Supplement: Supplementary file 1 [file ijms-27-06387-s001.zip › KIF21A Suppelementary files/KIF21A supplementary figures/Supllementary figure 2.tif]

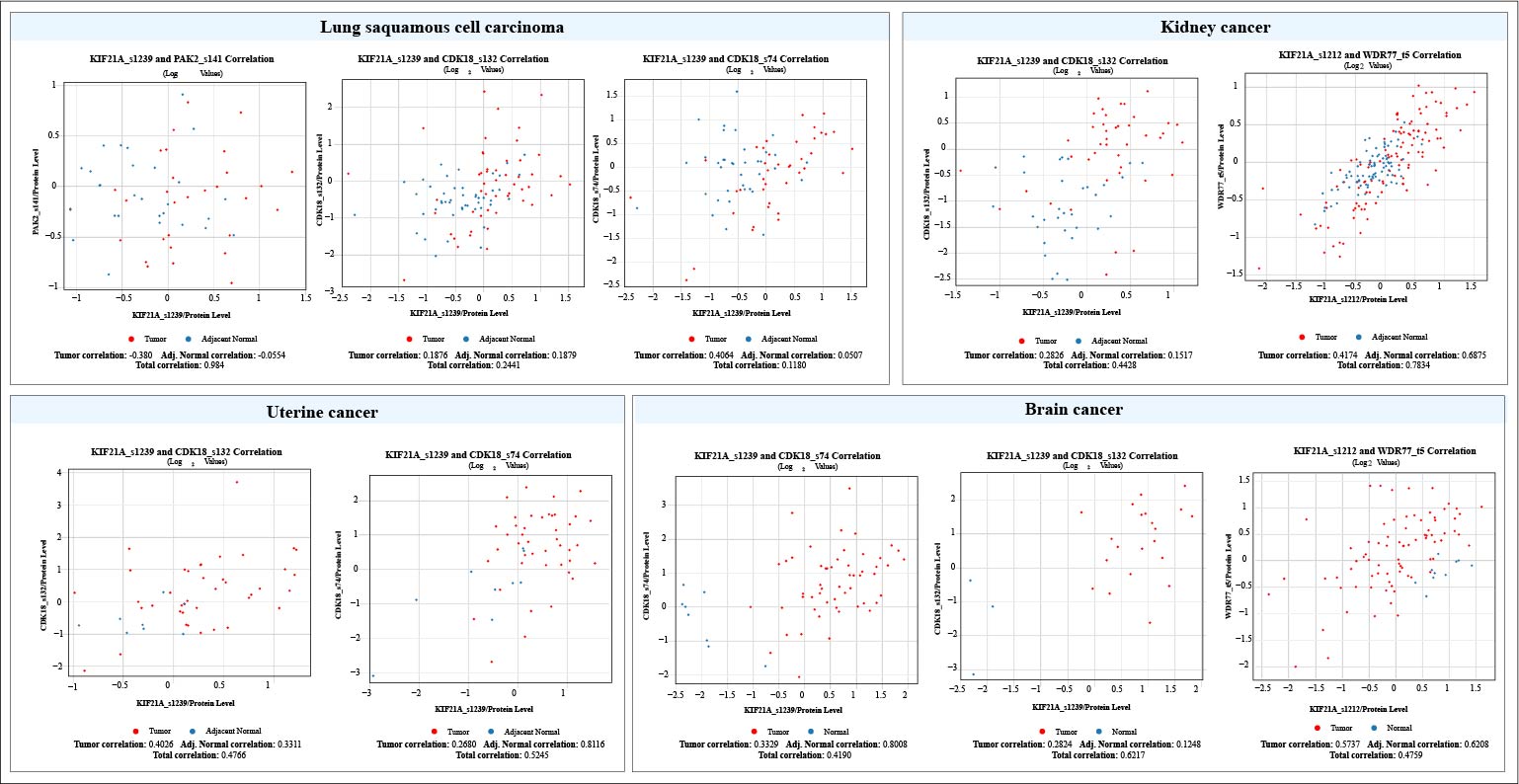

Supplement: Supplementary file 1 [file ijms-27-06387-s001.zip › KIF21A Suppelementary files/KIF21A supplementary figures/Supplementrary figure 1.jpg]
